# The Systemic Immune Response to Collagen-Induced Arthritis and the Impact of Bone Injury in Inflammatory Conditions

**DOI:** 10.3390/ijms20215436

**Published:** 2019-10-31

**Authors:** José H. Teixeira, Andreia M. Silva, Maria Inês Almeida, Mafalda Bessa-Gonçalves, Carla Cunha, Mário A. Barbosa, Susana G. Santos

**Affiliations:** 1 i3S—Instituto de Investigação e Inovação em Saúde and INEB—Instituto Nacional de Engenharia Biomédica, University of Porto, 4200-135 Porto, Portugal; 2 Department of Molecular Biology, ICBAS—Instituto de Ciências Biomédicas Abel Salazar, University of Porto, 4050-313 Porto, Portugal

**Keywords:** rheumatoid arthritis, collagen-induced arthritis, microenvironment, inflammation, mesenchymal stem/stromal cells, bone injury, repair/regeneration

## Abstract

Rheumatoid arthritis (RA) is a systemic disease that affects the osteoarticular system, associated with bone fragility and increased risk of fractures. Herein, we aimed to characterize the systemic impact of the rat collagen-induced arthritis (CIA) model and explore its combination with femoral bone defect (FD). The impact of CIA on endogenous mesenchymal stem/stromal cells (MSC) was also investigated. CIA induction led to enlarged, more proliferative, spleen and draining lymph nodes, with altered proportion of lymphoid populations. Upon FD, CIA animals increased the systemic myeloid cell proportions, and their expression of co-stimulatory molecules CD40 and CD86. Screening plasma cytokine/chemokine levels showed increased tumor necrosis factor-α (TNF-α), Interleukin (IL)-17, IL-4, IL-5, and IL-12 in CIA, and IL-2 and IL-6 increased in CIA and CIA+FD, while Fractalkine and Leptin were decreased in both groups. CIA-derived MSC showed lower metabolic activity and proliferation, and significantly increased osteogenic and chondrogenic differentiation markers. Exposure of control-MSC to TNF-α partially mimicked the CIA-MSC phenotype in vitro. In conclusion, inflammatory conditions of CIA led to alterations in systemic immune cell proportions, circulating mediators, and in endogenous MSC. CIA animals respond to FD, and the combined model can be used to study the mechanisms of bone repair in inflammatory conditions.

## 1. Introduction

Rheumatoid arthritis (RA) is an autoimmune condition, characterized by symmetrical joint inflammation, that affects approximately 1% of the world’s population [1]. RA is characterized mainly by synovium hyperplasia and a joint destruction process. In this scenario, immune cells and the inflammatory microenvironment that they create in affected joints are key components in the pathophysiology of RA. Moreover, it is well-described that during the inflammatory stages of the disease, extra-articular manifestations are common, which involve other tissues or organs [2].

RA patients are at risk of systemic complications and several co-morbidities, including osteoporosis and frequent vertebral and hip fragility fractures [3,4]. The incidence rate of overall fractures in RA patients is 33 per 1000 person-years [5], and the risk is increased with disease activity and associated with overexpression of pro-inflammatory cytokines that can disturb the bone remodeling process [4,6,7].

Over the last decades, animal models—especially the rodent models—have been crucial tools for understanding the biologic process of RA [8], and their use can aid in developing new therapeutic strategies for fracture healing in RA inflammatory conditions. Collagen induced-arthritis (CIA) animal models have been one of the most widely used models in RA research. Originally described by Trentham [9], CIA is a reproducible animal experimental model of RA [10,11]. In fact, the similarity to human RA regarding the disease clinical, histological, and immunological signals—including high articular levels of inflammatory cytokines—like tumor necrosis factor-α (TNF-α) [12,13], make it an invaluable model to study the pathologic process and to search new therapeutic strategies [14,15,16]. The response to rat CIA has been reported to involve macrophages, T and B lymphocytes, and mediators such as TNF-α, Interleukin (IL)-1β, IL-6, and IL-17 [17]. Nonetheless, the systemic response in this model has not been well characterized so far.

Importantly, current RA treatments do not promote joint repair, and several efforts are being made to develop new therapies, especially based in cell approaches using mesenchymal stem/stromal cells (MSC) [18,19]. MSC are multipotent progenitor cells with the potential to differentiate into mesenchymal lineage tissues (e.g., bone, cartilage, and adipose tissue), described to have immunomodulatory roles [20], being capable of recruiting different cell types and promoting tissue repair [21]. The transplantation of MSC has been reported to ameliorate or delay RA onset in CIA animals, partially mediated by inflammatory signaling suppression [22,23], and thereby reducing joint swelling and destruction [24,25]. Although the available evidence supports the use of MSC transplantation as a cell-based strategy in CIA animals, data on the biology of endogenous CIA animals-derived MSC in basal conditions is scarce. Moreover, the impact of the systemic inflammatory condition on biological properties of endogenous MSC has not been explored yet.

Herein, we propose CIA as a reliable model to study bone regeneration in inflammatory conditions, and additionally we investigate the effect of RA induction on the biological behavior of endogenous MSC as crucial cells involved in repair/regeneration.

Our results have shown that the combination of the two models is feasible and that CIA animals respond to the bone injury with a significant increase of systemic myeloid cells number and their co-stimulatory molecules (CD40 and CD86) expression, accompanied by increased levels of IL-13, IL-2, and IL-6 in plasma. The systemic inflammatory environment created by the arthritis induction leads to decreased metabolic activity and proliferation of CIA-derived MSC, and increased differentiation capacity determined by the expression of osteogenic (runt-related transcription factor 2 (RUNX2) and alkaline phosphatase (ALP)), and chondrogenic (aggrecan (ACAN)) markers in basal conditions.

## 2. Results

### 2.1. Collagen-induced arthritis (CIA) as a Model to Study Bone Injury in Inflammatory Conditions

**Figure 1:**
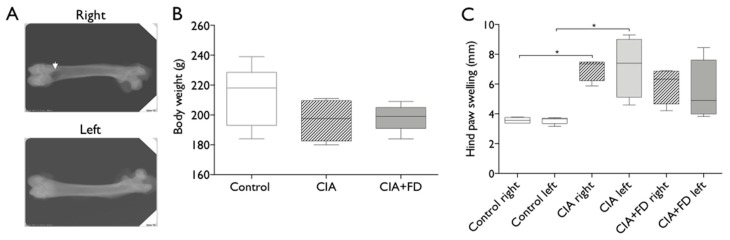
Combination of collagen-induced arthritis (CIA) model with femoral defect (FD). CIA was induced and allowed to develop for 21 days, before a cylindrical defect was performed in the femur of a half of the CIA animals to combine both animal models (CIA+FD) (**A**) X-ray of cylindrical defect performed on the right femur (white arrow indicates the defect site) of CIA animals at day 3 after surgery. (**B**) Body weight evaluation 3 days after bone defect. (**C**) Measurements of paw swelling in both paws (right and left) of control, CIA and CIA+FD, 3 days after bone injury. Box plots represent min-to-max distribution of *n* = 4 to 5 animals per group. * *p* < 0.05, determined by Kruskal–Wallis test and Dunn’s multiple comparisons test.

First, we established if the CIA model in the rat would be suitable to study the response to bone injury under inflammatory conditions by inducing it in otherwise healthy female Wistar rats and performing a critical size bone defect at day 21 after CIA induction (Figure 1).

Arthritis induction was effective in all animals, with macroscopic evidences of erythema and swelling in hind paws, and significant increases in swelling after day 14 when compared to the non-immunized/control animals (Figure S1A,B). The arthritis index score increased along the monitoring time until hitting a plateau between day 17 and 21 (Figure S1C).

At day 21 after immunization, a group of CIA animals with evident signals of arthritis were subjected to a cylindrical femoral bone defect (FD), as illustrated in the x-ray of Figure 1A, and followed up to three days post-injury. The combination of CIA with FD (CIA+FD) did not compromise animal welfare beyond the impact of CIA itself, with CIA+FD animals recovering well from surgery and keeping to a similar body weight as the control and CIA groups (Figure 1B). Hind paw swelling of CIA+FD animals showed a slight decrease at day 3 post-injury, albeit not significant (Figure 1C).

### 2.2. The Impact of CIA and Bone Injury in Secondary Lymphoid Organs

**Figure 2:**
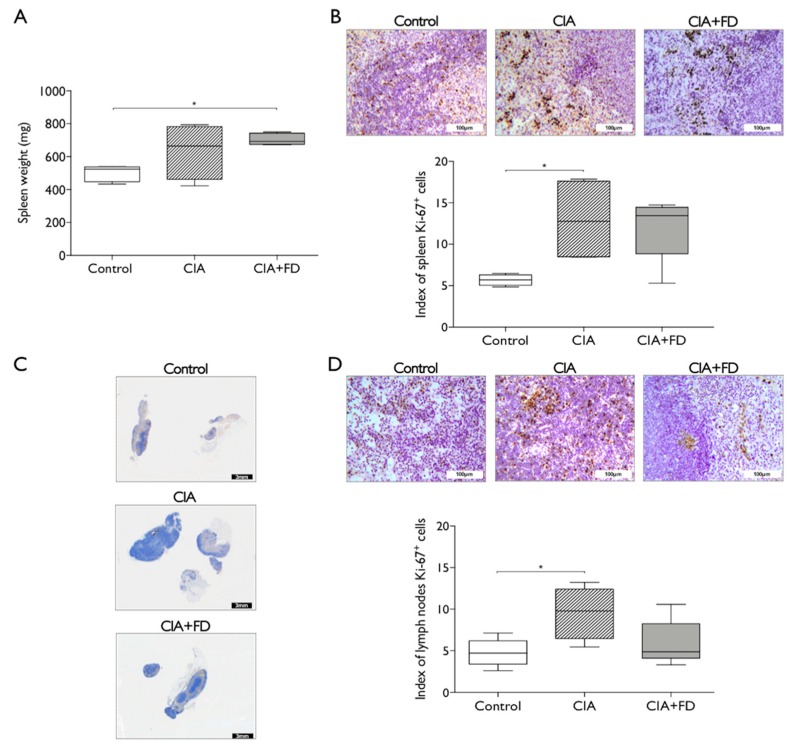
Arthritis induction and the combination with femoral defect promotes alterations in rat secondary lymphoid organs. (**A**) Spleen weight from control, CIA, and CIA+FD animals. (**B**) Representative spleen paraffin-section showing immunohistochemistry (IHQ) staining for the proliferation marker Ki-67, and quantitative staining evaluation across different sections of all animals. (**C**) Draining lymph nodes general view of histological analysis. (**D**) Higher magnification showing Ki-67 IHQ staining, and quantitative staining evaluation across different sections of all animals. Box plots represent min-to-max distribution of *n* = 4 to 5 animals per group. * *p* < 0.05 determined by Mann–Whitney test. Scale bar: 3 mm (black), 100 µm (white).

Next, we wanted to ascertain if CIA and its combination with a bone defect correlated with acute systemic changes in secondary lymphoid organs. Spleen from CIA and CIA+FD groups were collected three days after bone injury and found to be enlarged when compared to control, with a significant increase in weight for CIA+FD animals (Figure 2A). Cell proliferation was assessed by Ki-67 staining, and was also increased in both CIA and CIA+FD groups, but a statistically significant increase was only observed for the CIA group (Figure 2B). Lymph nodes draining the hind paws were also collected and found to be enlarged in CIA and CIA+FD groups (Figure 2C). Cell proliferation in lymph nodes was quantified across the different animals and found significantly increased in CIA animals when compared to controls (Figure 2D). Overall, these results support that spleen and lymph nodes are responding to CIA induction with increased cell proliferation.

**Figure 3:**
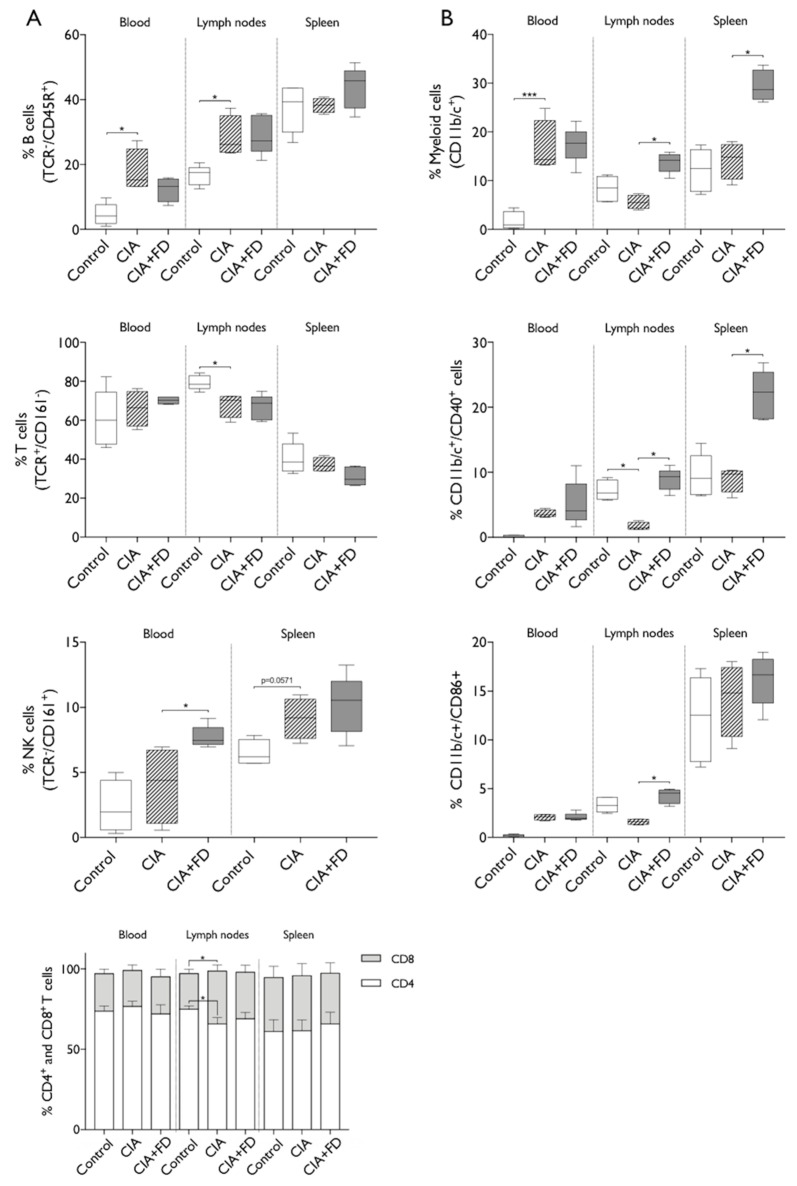
CIA and CIA+FD lead to alterations in systemic immune cell proportions. The percentages of lymphoid and myeloid populations at systemic level were analyzed in blood, draining lymph nodes and spleen of control, CIA, and CIA+FD animals, by multicolor flow cytometry. (**A**) Percentage of main lymphoid populations: B cells (TCR^−^/CD45^+^), T cells (TCR^+^/CD161^−^), NK cells (TCR^−^/CD161^+^), and CD4 (TCR^+^/CD4^+^) and CD8 (TCR^+^/CD8^+^) T cells. (**B**) Myeloid cells (CD11b/c^+^), and their activation status as determined by co-expression of the co-stimulatory markers CD40 and CD86. Box plots represent min-to-max distribution of *n* = 4 to 5 animals per group. * *p* < 0.05, *** *p* < 0.001 determined by Kruskal–Wallis test and Dunn’s multiple comparisons test.

To further determine the impact of CIA and CIA+FD in systemic immune cell populations, we performed multicolor flow cytometry analysis of cells from blood, draining lymph nodes and spleen. The gating strategy is illustrated in Supplementary Figure S2. Results obtained are summarized in Figure 3 and show significant changes in the proportion of lymphoid and myeloid cells in blood, draining lymph nodes and spleen. Concerning the lymphoid lineage populations (Figure 3A), we observed a significant increase in the percentage of B lymphocytes in blood and lymph nodes from CIA animals relative to control animals, but no differences were observed in spleen. T cell percentage was significantly decreased in lymph nodes of CIA animals, while Natural Killer (NK) cells were increased (*p* = 0.0571) in the spleen of those animals. The proportions of CD8^+^ and CD4^+^ T cells were similar between all groups in blood and spleen, but significant differences were observed in CIA lymph nodes. Results showed an increased percentage of CD8^+^ and decreased CD4^+^ T cells in CIA animals when compared to control animals. Bone injury (CIA+FD) did not induce further significant changes in lymphoid cell proportion in CIA animals at three days post-injury, except for a significant increase in NK cells in blood at day 3 after bone defect.

Analyzing the myeloid cells (Figure 3B) revealed a significantly higher percentage of myeloid cells in blood of the CIA group, which did not change with bone injury. Conversely, in draining the lymph nodes and spleen, no increases were observed in the CIA group, but there were significant increases in percentages of myeloid cells 3 days after bone injury. To further explore these results, expression of co-stimulatory molecules CD40 and CD86 was analyzed to investigate myeloid cell activation. Cells in circulation from CIA animals present increased the percentage of cells expressing each molecule, albeit not significant, while lymph node cells showed a significant decrease of the percentage of myeloid cells expressing CD40, with CD86 following the same tendency. Importantly, 3 days after bone injury, myeloid cells showed increased expression of both co-stimulatory molecules CD40 and CD86, in lymph nodes, and for CD40 also in spleen, when compared to the CIA group. Overall, these evidences suggest a persistent systemic immune response in CIA animals that are nonetheless still able to respond to an acute injury, particularly increasing their myeloid cells proportion and activation.

### 2.3. The Impact of CIA and Bone Defect in Circulating Inflammatory Mediators

**Figure 4:**
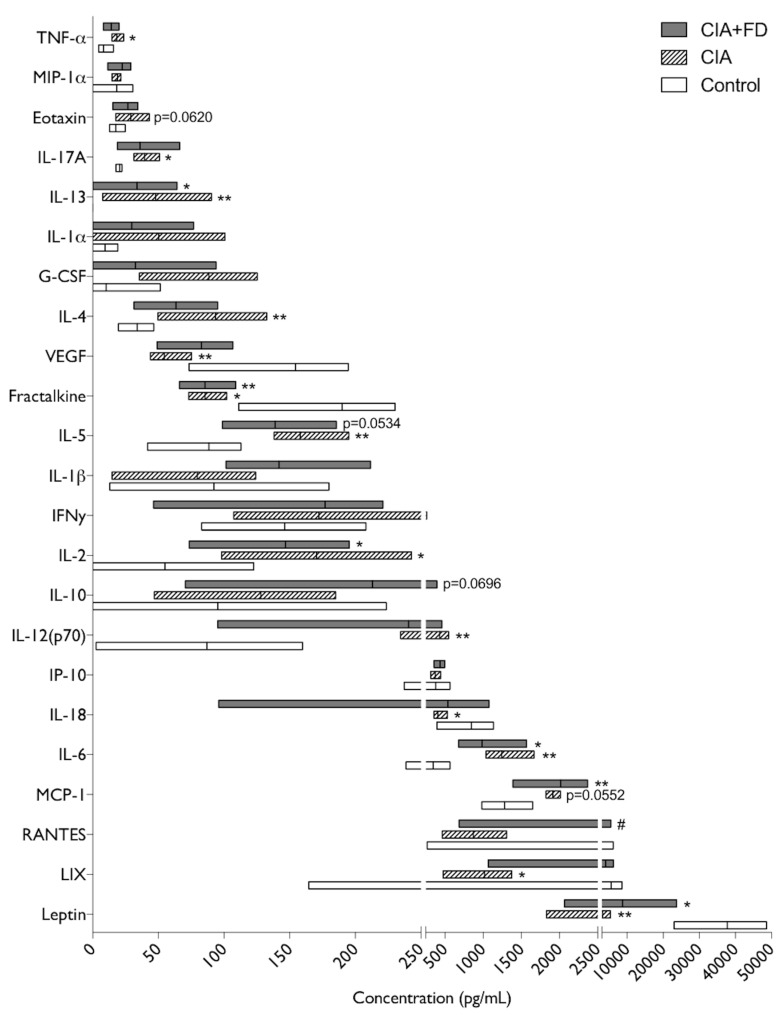
Changes in plasma cytokine and chemokine profile in CIA and CIA+FD. Quantitative results of the multiplex cytokine/chemokine array performed for all animals in each group. Box plots represent min-to-max distribution of *n* = 4 to 5 animals per group. * *p* < 0.05, ** *p* < 0.01 in relation to control group, and # *p* < 0.05 relatively to CIA group, determined by Kruskal–Wallis test and Dunn’s multiple comparisons test.

To further investigate the systemic inflammatory impact of CIA and the combination with bone injury, 27 chemokines and cytokines were quantified in plasma. From these, epidermal growth factor (EGF), growth-regulated oncogenes/keratinocyte chemoattractant (GRO/KC), Macrophage inflammatory protein-2 (MIP-2, CXCL2), and Granulocyte-macrophage colony-stimulating factor (GM-CSF) were out of the detection range for this assay. From the remaining 23 cytokines/chemokines detected, IL-13 was not detected in the control group, but was detected in the other two animal groups and thus was considered in the analysis. Results obtained are illustrated in Figure 4 and show that, relative to the control group, CIA animals had a significant upregulation of 8 molecules and a downregulation of 5. Among the significantly up-regulated mediators IL-13, IL-2, and IL-6 were also significantly up-regulated in the CIA+FD group, while TNF-α, IL-17 A, IL-4, IL-5, and IL-12 (p70) lost significance in the CIA+FD group. Nonetheless, the tendency for increase was maintained, and in the case of IL-5 it was close to statistical significance (*p* = 0.0534). Also, Monocyte chemoattractant protein-1/C-C motif chemokine ligand 2 (MCP-1/CCL2) and Eotaxin were close to statistical significance in CIA (*p* = 0.0552 and *p* = 0.0620, respectively), and MCP-1 was significantly up-regulated for the CIA+FD group. Regarding the downregulated mediators identified in CIA plasma, Vascular endothelial growth factor (VEGF), Fractalkine, IL-18, C-X-C motif chemokine 5 (CXCL5, LIX), and Leptin, only Fractalkine and Leptin levels were statistically significantly lower in the CIA+FD group, relative to control. The only molecule that showed a significant difference between the CIA and CIA+FD groups was the regulated on activation, normal T cell expressed and secreted (RANTES) chemokine, which was significantly downregulated in CIA when compared to CIA+FD, but neither group had a significant difference to the control group.

### 2.4. CIA Induction Impacts Endogenous MSC Biologic Behavior

To evaluate the impact of chronic inflammation on endogenous MSC we next isolated and culture bone marrow-derived MSC (BM-MSC) from CIA and control animals. The number of bone marrow cells recovered was similar between the CIA and control animal groups, and after selective culture and expansion, cells obtained were highly positive for the classical stromal markers CD29 and CD90, and did not express the haematopoietic marker CD45 (Supplementary Figure S3). The bone marrow isolated cells showed the ability to differentiate into the chondrogenic, osteogenic, and adipogenic lineages (Supplementary Figure S4). All together, these data confirmed the successful isolation of MSC from bone marrow of both CIA and control animals.

**Figure 5:**
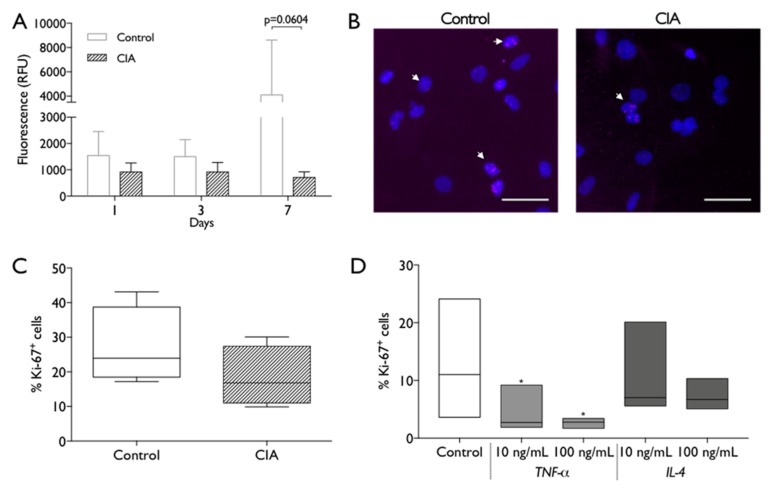
MSC metabolic activity and proliferation capacity. (**A**) Metabolic activity was measured at 1, 3 and 7 days of MSC culture by resazurin assay. RFU: relative fluorescence units. Statistical differences were evaluated by 2-way ANOVA followed by Turkey’s multiple comparison test (**B**) Representative images of nuclei (Dapi, blue) and Ki-67 immunostaining (pink, white arrows) of control and CIA-derived MSC after 2 days in culture. Scale bar: 50 µm. (**C**). Percentage of Ki-67^+^ control and CIA-derived MSC, across different experiments. Statistical differences were evaluated by Mann–Whitney test. (**D**) Percentage of Ki-67^+^ control- derived MSC after 2 days in absence/presence of 10 ng/mL or 100 ng/mL of TNF-α or IL-4. Box plots represent min-to-max distribution of *n* = 4 to 5 animals per group. * *p* < 0.05 by Friedman test followed by Dunn’s multiple comparisons test.

BM-MSC were then used to evaluate the impact of the cells source microenvironment on their biological properties and behavior. CIA and control BM-MSC were metabolic active along 1, 3, and 7 days of culture, but CIA-MSC showed lower metabolic activity that did not increase along time, when compared to control cells (Figure 5A). In line with these results, the Ki-67 fluorescent immuno-staining analysis also showed a tendency for reduced proliferation of CIA-MSC (Figure 5B,C). To access if exposure to the inflammatory microenvironment could be conditioning, MSC proliferation control cells were cultured in the presence of TNF-α or IL-4. Interestingly, even low doses of TNF-α produced significant reductions in the percentage of proliferating cells, while IL-4 had no significant impact (Figure 5D).

**Figure 6:**
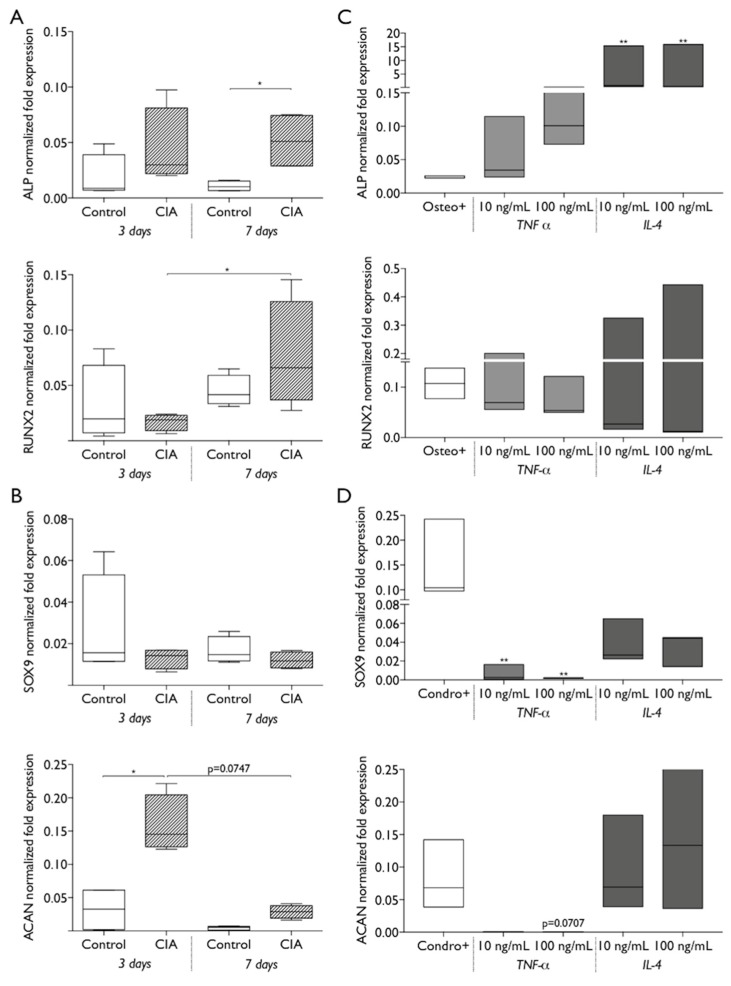
The capacity of MSC differentiation is affected by arthritis induction. The differentiation of control and CIA-MSC were evaluated in absence of chemical inductors, at 3 and 7 days of culture by reverse transcription-quantitative polymerase chain reaction (RT-qPCR) for (**A**) osteogenic marker genes ALP and RUNX2, and (**B**) chondrogenic differentiation genes SOX9 and ACAN. Box plots represent min-to-max distribution of *n* = 2 to 3 different animals per group, in 2 independent experiments. * *p* < 0.05 by Kruskal–Wallis test followed by Dunn’s multiple comparisons test. The effect of 10 ng/mL or 100 ng/mL of TNF-α and IL-4 in the differentiation of control MSC, were evaluated in basal conditions at 3 days by RT-qPCR for (**C**) osteogenic and (**D**) chondrogenic markers. Cells cultured with osteogenic and chondrogenic inductors were used as positive controls for osteogenic (osteo+) and chondrogenic (chond+) differentiation. * *p* < 0.05, ** *p* < 0.01 determined by Friedman test followed by Dunn’s multiple comparisons test.

The endogenous differentiation ability of BM-MSC from CIA and control animals was evaluated at 3 and 7 days of culture, in the absence of chemical and molecular inductors. The results obtained showed that CIA animals-derived MSC had increased expression of osteogenic genes, with significantly higher ALP expression, relative to control cells, at 7 days, and a significant increase in RUNX2 expression from 3 to 7 days in CIA-MSC, which was not observed in control-MSC (Figure 6A). When analyzing chondrogenic differentiation capacity of CIA-MSC in basal conditions, results indicate a significantly higher expression of ACAN at 3 days compared to control cells, and that significantly decreased by day 7, while expression of SOX9 did not show significant differences between the cells of CIA and control animals, or along time (Figure 6B). Then we explored if exposure to cytokines could impact MSC differentiation in the absence of chemical induction. Results obtained show that exposure to TNF-α or IL-4 increased ALP gene expression with IL-4 exposure producing significant increases, even when compared to the positive control, using chemical inductors (Figure 6C). Interestingly, IL-4 exposure led to increased ACAN expression, to levels similar to those obtained with chondrogenic induction and for CIA- MSC, while TNF-α did not lead to the increases observed for MSC from CIA animals (Figure 6D).

Taken together, these results indicate that exposure to hallmark arthritis cytokines like TNF-α can partially mimic the CIA impact on MSC.

## 3. Discussion

In the current study, arthritis was induced in all animals and reached a plateau in its score by 17 to 21 days after CII-IFA (Collagen type II-Incomplete Freund’s adjuvant) immunization. This is in line with previous reports on this model [26]. Histologically, our data shows hind paws swelling and inflammation in digital joints, visible by the infiltration of inflammatory cells and hyperplasia of the synovial membrane, which constitute hallmarks of human RA pathology [27] and have been previously reported in CIA animal model [11,28,29]. The CIA model was combined with a critical size femoral bone defect model in a load-bearing site without the need for fixation material [30] and without significant acute implications for animal welfare and paw swelling. Enlargement of secondary lymphoid organs was similar between CIA and CIA+FD at 3 days post-bone injury, albeit slightly decreased percentages of proliferating cells in CIA+FD were observed. This is in line with previous work in the CIA model, such as in the report by Ibraheem where the injection of different collagen types combined with adjuvant to induce arthritis in rats led to enlarged lymph nodes with histologic alterations [31] and the reports of splenomegaly in the most severe states of adjuvant-induced arthritis [11,32]. Also, a previous report induced a mid-shaft femur fracture with fracture fixation in the CIA rat model and showed delayed healing in CIA animals, but the authors do not analyze inflammation in their study [33]. In fact, the current study is, to the best of our knowledge, the first one addressing the interplay between inflammation and bone injury in these RA models. 

To determine the systemic immune cell response to CIA induction and the combination with bone injury, we evaluated the main immune cell populations that are the key cell players involved in the development and maintenance of the inflammation in CIA model [10], in blood and secondary lymphoid organs. Our results show a significant increase in B cells in blood and draining lymph nodes of CIA animals and also increased myeloid cells in blood. This change in proportions may suggest that these cells migrate from the blood to the inflamed tissues. A previous report in the mouse CIA model described similar increases in the percentage of B cells, which they correlate with increased anti-collagen antibodies in serum [34]. Activated B cells have been reported to produce anti-collagen antibodies that bind to cartilage collagen and form an immune complex, generating a localized inflammatory response and attracting monocytes, granulocytes, and T cells to the joint [10,17]. 

Interestingly, CIA animals responded to the bone injury with significant increases in myeloid cells and their activation, particularly in secondary lymphoid organs. Myeloid cells are important regulators that contribute to the perpetuation of inflammation in RA [35]. Moreover, in a murine model of early RA, increased myeloid dendritic cells were reported in draining lymph nodes before the appearance of joint histological changes, indicating their role in the early stage of murine RA [36]. A significant decrease in T cell proportions was found in lymph nodes of CIA rats, accompanied by a decrease in CD4/CD8 ratios. These data may suggest that T cells are attracted to the joints, but this requires further confirmation. The immune response mediated by T cells is described as crucial for the development of mouse [10,17] and rat CIA, with athymic rats being resistant to CIA induction, but T cell transfer alone was reported to have induced only a mild response [10]. The role of NK cells in RA and CIA is not yet clear, with some evidence that they may contribute to [37,38] or protect against [39] inflammation. Here, we did not observe differences in the percentage of NK cells in peripheral blood as described in a previous study in CIA mice [34], but observed an increase in the CIA+FD group. The same work showed a decrease in splenic NK cells proportion at day 37, different from the increasing tendency that was observed in the current study at day 21 after CIA induction. Interestingly, the changes observed here in systemic immune cell proportions are different from those previously observed in the same model of bone injury, but in healthy animals at day 6 post-surgery [40]. This might be explained by the inflammatory conditions, but also by different times post-injury, as in the same model in healthy animals we observed an acute response at day 3, and resolution of that response at day 14 post-surgery [41].

In the current study, a broad quantitative analysis of plasma cytokines/chemokines comparing control, CIA, and CIA+FD animals was performed. We found increased levels of the hallmark cytokine TNF-α in the plasma of CIA animals. High TNF-α levels in affected joints or in peripheral blood serum are strongly associated with RA disease severity [42,43]. This pro-inflammatory cytokine was described in different RA animal models [43,44], including the transgenic mouse expressing human TNF-α [45] and the CIA model [12,13]. TNF-α has also been reported as accelerating arthritis signals when injected into animals [43]. In response to TNF-α, RA synovial fibroblasts are described to produce MCP-1/CCL2, which was also found increased in the plasma of CIA animals in the current study, particularly after bone injury. MCP-1 is a potent chemokine that mediates monocyte ingress and activation in joints [46]. This was clearly demonstrated in vivo by the injection of a rabbit model with MCP-1/CCL2, resulting in a marked macrophage infiltration of the affected joints [47]. Macrophages have been previously described as a major source of MCP-1/CCL2 [48]. In our results, CD68^+^ macrophages were also found in the inflamed synovium.

Levels of IL-17, IL-2, and IL-5 were also increased in CIA animals, and maintained the same tendency (albeit not always significant) in the CIA+FD group. These cytokines are related to the T cell function and response in vivo. Systemic IL-17 levels intensify RA and induce a chronic and erosive form of the disease [49]. A recent report in CIA model in Dark Agouti rats demonstrated that the Th-17 response in draining lymph nodes is greater in females than in males, correlating the Th-17/Treg (regulatory T cell) axis with sexual differences in CIA severity [50]. IL-2 is a positive regulator of T cell proliferation, and administration of IL-2 can mediate a pro-inflammatory effect on CIA mice, increasing the animals’ arthritic scores [51]. A recent report shows that IL-2-Anti-IL-2 monoclonal antibody immune complexes can inhibit CIA by increasing Treg cell functions [52]. IL-5 is a cytokine with pleiotropic effects on target cells, including eosinophils and B cells, inducing cell proliferation, survival, and differentiation [53], its role in RA remains unknown, but a recent work found elevated serum levels of IL-5 in 59% of seropositive RA patients [54]. Also, it was reported that lymph node cells isolated from CIA-susceptible mice lines showed a higher number of IL-5 producing cells in culture than cells from resistant mice [55]. Fractalkine was found downregulated in CIA animals with and without bone injury, which is not in agreement with reports indicating amelioration of arthritis symptoms and joint destruction when Fractalkine is suppressed [56,57]. Also, Leptin was found significantly reduced in the plasma of CIA and CIA+FD animals, but its role in arthritis is still controversial. In the mouse CIA model, Leptin injection has been reported to worsen disease via Th-17 cells [58], but has also been reported as anti-inflammatory when collagen-antibody-induced arthritis is induced in mice overexpressing Leptin [59]. Also, its levels have been reported as increased in other models of RA and in patient’s plasma, but reduced at the joint level, indicating its consumption [60]. RANTES (or CCL5) was the only molecule that showed a significant difference between the CIA and CIA+FD groups. A recent report on bioinformatic analysis of human RA gene expression data indicates that CCL5 might have a negative impact on the development of RA [61], while another recent report in mouse indicates that peritoneal levels of several CC chemokines, including CCL5, could be related with mouse strain susceptibility to experimental arthritis [62]. However, as in our study, neither group had a significant difference to control, and so the biological meaning of this result remains unclear.

Although the use of MSC as therapeutics for RA is being intensely studied, the effect of CIA induction and its inflammatory milieu on the biological behavior of endogenous bone marrow-derived MSC had not been explored so far. Growing evidence indicates that bone marrow is actively involved in the RA disease process. In RA patient samples, the interaction between synovial inflammatory tissue and bone marrow reportedly results from the disruption of cortical bone leading to bone marrow invasion [63]. Furthermore, previous work described in vivo a significant enlargement of vascularized bone canals that link the bone marrow and the synovium tissue, in joints with synovial hyperplasia [64]. Also, it has been suggested that bone marrow-derived immature mesenchymal cells may migrate through the joint space to replace synovial cells, similar to what happens with inflammatory cells [65,66]. In fact, even if bone marrow is not primarily affected, this interaction, together with the aberrant cytokine production, can potentially affect the biological behavior, namely the survival and proliferation of bone marrow cells [67].

The current work shows that culture-expanded MSC from CIA animals were morphologically and immunophenotypically similar to controls, expressing the characteristic mesenchymal markers (Figure S1C). Nevertheless, the metabolic activity and percentage of proliferative cells were lower in the CIA-MSC cultures. This had not been described in RA animal models thus far, but agrees with what was described in cells derived from RA patients [68,69]. RA-MSC display proliferation defects in culture without significant differences in the percentages of apoptotic cells [68,69], and high levels of the cell cycle inhibitor p21, which can mediate the defects in cell growth and replicative capacity [69]. Mice treated with anti-TNF-α at an early CIA stage showed a reduction in the number of MSC in the bone marrow and synovium [64]. Conversely, a previous report described that the proportion of CD34^+^ bone marrow progenitor cells increase after the anti-TNF-α treatment in RA patients, with the number of bone marrow mononuclear cells in clonogenic assays being higher when compared to the pretreatment values [67]. In our study, a significant reduction in the percentage of proliferative MSC was observed in control cells exposed to TNF-α, an important pro-inflammatory cytokine found increased in the plasma of CIA animals. This reduction in healthy MSC proliferation in the presence of TNF-α could have consequences for MSC-based therapies for RA.

Our results show increased osteogenic gene expression of CIA-MSC in basal conditions when compared to control MSC. Contrasting data has been reported on the osteogenic and chondrogenic potential of MSC isolated from RA patients. Previous reports indicate that ALP expression was comparable between the cells from RA and healthy donors [68,69], and that RA MSC do not differ significantly from the normal cells on their chondrogenic differentiation [70]. MSC differentiation in the CIA animal model has been analyzed in response to the chemical/molecular stimulation protocols commonly used in vitro, but not in basal conditions, and further detailed analysis is still required. Previous work showed that control and CIA-derived mouse BM-MSC respond to osteogenic stimulation with similar increases in RUNX2 and ALP gene expression [71]. In our work the cells were isolated from the same site and from animals with the same age and sex. Thus, our results support that the alterations in proliferative and differentiation capacities are not site or age-dependent, but are likely associated with the disease condition of the animals. We also explored if CIA conditions could be mimicked by exposure to TNF-α and observed an increase, albeit not statistically significant in ALP gene expression. This agrees with previous reports showing that exposure to these concentrations of TNF-α impairs MSC proliferation/survival [72]. The same authors also found impaired MSC response to osteogenesis induction upon exposure to these concentrations of TNF-α, but the response of the cells in the absence of osteogenic induction was not analyzed. In the RA model of human TNF-α transgene, previous reports showed the deleterious effects of high levels of this cytokine on fracture repair [45], and in the CIA model fracture healing was also impaired [33]. Further studies are required to analyze if such impairments are related to changes in MSC proliferation and differentiation, and the role of chronic inflammation in bone repair [72].

In conclusion, arthritis induction creates a systemic inflammatory environment, with changes in immune cells and circulating cytokine/chemokine levels. Also, bone marrow-derived MSC isolated from CIA animals display impaired proliferation and increased differentiation in basal conditions. Further studies are needed to clarify the relation between exposure to inflammatory cytokines like TNF-α and MSC phenotype impairments, and their consequences for bone repair in RA. Furthermore, the current work supports the viability of using the CIA model to investigate the mechanisms of bone repair/regeneration in chronic inflammatory conditions, and the development of therapeutic strategies more appropriate to treat bone fractures in RA patients.

## 4. Materials and Methods

### 4.1. Bovine Collagen Type II Emulsion Preparation

Bovine collagen type II (CII, 2 mg/mL, Chondrex, Redmond, WA, USA) was added in a 1:1 proportion of incomplete Freund’s adjuvant (IFA, Chondrex, Redmond WA, USA) and homogenized in an ice water bath, according to the manufacturer’s instructions. Then, the emulsion was kept at 4 °C until injection, without this period exceeding 1 h.

### 4.2. Collagen-Induced Arthritis (CIA) Rat Model

Animal procedures were performed in accordance with the ethical animal welfare and experimentation, and approved by the Portuguese official authority regulating laboratory animal sciences (DGAV). Fifteen female Wistar rats (7–8 weeks old) were purchased from Charles River Laboratories (Barcelona, Spain) and housed in a clean environment. Animals were randomly divided into CIA (*n* = 10) and healthy control (*n* = 5) groups. On day 0, CIA rats were immunized with 200 µL of CII-IFA emulsion (200 µg collagen/rat) with a 25-gauge needle via subcutaneous injection at the base of the tail. Seven days after, a booster injection of CII-IFA emulsion was administrated to ensure a high incidence and severity of arthritis. Animals were maintained under general isoflurane anesthesia during these interventions, and all the arthritis-inducing procedures were performed according to the guidelines of Chondrex and based in previous reports [9,15]. At day thirteen after CIA induction, one animal from CIA group was lost due to a problem of gastric obstruction.

### 4.3. Monitoring of Clinical Arthritis Development

The animal health condition and physical activity was monitored daily according to the recent guidelines of refinement in RA research [32]. Three times per week the animals were weighed, paw swelling was measured with digital caliper at the metacarpus level, and x-ray was performed. Each rat paw was scored individually according to signs of joints (interphalangeal, metacarpophalangeal, carpal and tarsal) inflammation, adapting the Chondrex arthritis scoring system: (0) normal, (1) mild redness and swelling of the ankle or digits, (2) moderate redness and swelling of ankle or wrist, (3) severe redness of the entire paw, (4) maximally inflamed limb with the involvement of multiple joints. The arthritis index was determined by the sum of the score of each front and hind paw with the total maximum of 16. 

### 4.4. CIA Rat Model Combined with Bone Injury Model

Twenty-one days after arthritis induction, a critical size femur defect was performed in 5 CIA rats, as previously described by our group [30]. Briefly, knees were shaved and disinfected, and then an incision was made in the skin and muscles surrounding the right femur were retracted. After lateral knee arthrotomy, a cylindrical defect (FD, 3 × 4 mm, diameter × depth) was created using a surgical drill (micromotor, K-control TLC 4965) in the anterolateral wall of the lateral condyle of the femur. Surgeries were performed under general anesthesia with volatile isoflurane. After surgery, analgesia was provided by subcutaneous administration of buprenorphine (0.05 mg/kg) twice a day. Three days after the bone injury, animals were euthanized for blood and organ collection.

### 4.5. Blood, Spleen and Lymph Nodes Collection and Processing

At day 24, animals were maintained under general anesthesia with volatile isoflurane and whole blood was collected by cardiac puncture into tubes containing anticoagulant citrate-phosphate-dextrose solution (Sigma-Aldrich Inc., St Louis, MO, USA). Blood was centrifuged, and plasma and buffy coats collected and processed as described previously [41]. Plasma was further centrifuged twice at 2500× *g* for 15 min and supernatant stored at −80 °C until further analysis. Collected buffy coats were diluted in phosphate buffered saline (PBS), overlaid on Lymphoprep (Axis-Shield Diagnostics, Dundee, Scotland) in a 1:1 ratio and centrifuged at 800× *g* for 30 min at RT to isolate peripheral blood mononuclear cells (PBMC). 

Animals were dissected for the collection of spleen and draining lymph nodes (inguinal and popliteal nodes). Spleen was clean from adjacent tissue and weighted, and then a half portion of spleen and lymph nodes were preserved in formalin 10% for further histology processing, whereas the remaining parts were used to isolate single cells for flow cytometry analysis, as described previously [41]. Briefly, spleen was digested with 1 mg/mL Collagenase I (Sigma-Aldrich Inc., St Louis, MO, USA), both spleen and lymph nodes were gently crushed, and strained to obtain cell suspensions. Red blood cells in spleen cell suspension were then lysed by incubation with NH_4_Cl 150 mM in Tris 10 mM. Obtained cells were washed with PBS. 

### 4.6. Histology and Immunohistochemistry Analysis

Animal hind paws were collected, fixed in formalin 10% for 48h, and then decalcified in 10% EDTA solution, for 1 month. Tissue was embedded in paraffin and sections of 4 µm thickness were cut and stained with hematoxylin and eosin (H&E). Spleen and draining lymph nodes were fixed for 24 h, paraffin embedded and 3 µm thickness paraffin sections were cut for H&E staining and immunohistochemistry (IHQ).

Expression of proliferation marker Ki-67 and macrophage marker CD68 was assessed by IHQ. NovolinkTM Polymer Detection Kit (Leica Biosystems, Newcastle, UK) was used following the manufacturer’s instructions. Antigen retrieval was performed by sections incubation in citrate buffer (98 °C, citrate buffer 10 mM, pH 6.0). After endogenous peroxidase neutralization, permeabilization with Triton X-100 0.3% was performed for Ki-67 immunostaining. Sections were incubated with Block protein solution and then in primary antibodies: anti-Ki-67 (1:50, clone SP6, ThermoFisher Scientific, Waltham, MA, USA) and anti-CD68 (1:100, clone ED1, Bio-Rad Laboratories, Irvine, CA, USA) for 45 min at RT. Positive staining was revealed after 30 min of incubation with NovoLinkTM Polymer and 5 min of incubation with peroxidase-substrate solution DAB. Finally, sections were counterstained with hematoxylin and slides mounted. Sections incubated without primary antibody were used as negative controls. Representative images of positive staining were taken using Olympus CX31 light microscope (20× objective). Nine images per slide were analyzed using Fiji software, and the number of nuclei Ki-67^+^ and Ki-67^−^ nuclei were counted. Proliferation index was calculated as the ratio of Ki-67^+^ nuclei to the total number of nuclei counted.

### 4.7. Flow Cytometry Analysis

Cells from blood, spleen, and lymph nodes were incubated with 1 µL per sample of mouse anti-Rat CD32 (clone D34-485, 20 µg/mL, BD biosciences, San Jose, CA, USA) diluted in staining buffer (0.5% bovine serum albumin (BSA) and 0.01% sodium azide in PBS), to prevent unspecific bindings. Then, cells were immunostained for 30 min on ice for the presence of T cells, B cells, NK cells, and myeloid cells by the following surface antibodies: anti-TCR-PerCP (clone R73, 4 µg/mL), anti-CD4-APC (clone OX-35, 4 µg/mL), anti-CD8a-V450 (clone OX-8, 8 µg/mL), anti-CD45R-PE (clone His24, 8 µg/mL), anti-CD161a-FITC (clone10/78, 2 µg/mL), anti-CD11b/c-PE-Cy7 (clone OX-42, 1.6 µg/mL), anti-CD40-FITC (clone HM40-3, 40 µg/mL), anti-CD86-PE (clone 24F, 16 µg/mL) all from BD biosciences. Cells stained with the corresponding isotype antibodies were used as control. Cells were washed 4 times in PBS and fixed with paraformaldehyde 1%. Samples were analyzed on a FACSCanto flow cytometer (Becton Dickinson), with acquisition of 10.000 events in gate for each sample, and data analyzed with FlowJo software.

### 4.8. Plasma Cytokine Quantification

Plasma cytokines were quantified at Eve Technologies Corp. (Calgary, AB, Canada) using the Rat Cytokine Array/Chemokine Array 27-Plex Discovery Assay. Plasma samples, 30 µL, were diluted twice in PBS prior analysis, according to the manufacturer’s indications. The sensitivity of the assay for the markers analyzed ranged from 0.1 to 15.7 pg/mL.

### 4.9. Isolation of MSC, Primary Culture and Phenotypic Characterization

Rat MSC were isolated form CIA and control femurs, as previously described [41]. Briefly, bone marrow was flushed with PBS and bone debris were removed by filtering the cell suspension through a 100 µm cell strainer. After red blood cells lysis, cells were counted and seeded in minimum essential medium alpha modification (α-MEM) supplemented with 10% MSC-qualified fetal bovine serum (ThermoFisher Scientific, Waltham, MA, USA MA, USA) and 1% penicillin G-streptomycin (P/S; Invitrogen, Carlsbad, CA, USA), at 10^6^/100 mm plate. MSC were selected by adherence to plastic surface, expanded and cryopreserved for forward use. Isolated and cultured MSC identity was confirmed by multicolor flow cytometry based on classical cell surface markers CD29^+^ (anti-CD29-APC, HMβ1-1, BioLegend, San Diego, CA, USA), CD90^+^ (anti-CD90-PE, MRC OX-7, Immunotools, Friesoythe, Germany) and CD45^−^ (anti-CD45-FITC, MRC OX-30, Immunotools, Friesoythe, Germany) staining, and by ability of cells to differentiate into the osteogenic, chondrogenic and adipogenic lineages. Cells from CIA and control animals were maintained in a humidified incubator, at 37 °C and 5% CO2, and used for experiments between passages 4 and 8.

### 4.10. MSC Metabolic Activity and Proliferation Assays

MSC were seeded in a 96-well plate at 2000 cells/well and allowed to reach 40% to 60% confluence. Then, metabolic activity and proliferation assays were performed. Metabolic activity was evaluated by resazurin assay, incubating cells with 10% Alamar blue (ThermoFisher Scientific, Waltham, MA, USA) in MSC culture conditions for 4 h. Fluorescence was measured (530 nm excitation and 590 nm emission) in a Synergy HT Multi-Mode Microplate Reader (BioTek Instruments, Winooski, VT, USA). This procedure was repeated at 1, 3 and 7 days of culture in five replicates. Samples without cells were used as negative controls. 

To determine the MSC proliferation index, cells were fixed after 48 h in culture in absence/presence of TNF-α or IL-4 (Immunotools, Friesoythe, Germany) at 10 ng/mL and 100 ng/mL. After permeabilization step with Triton X-100 0.1% and block with 5% BSA, cells were labelled for Ki-67 (1:150; clone SP6, ThermoFisher Scientific, Waltham, MA, USA) followed by AlexaFluor647-conjugated secondary antibody, and nuclei stained with DAPI 1 µg/mL. Cells incubated with secondary antibody only were used as a negative control. Immunofluorescence images were acquired in the IN Cell Analyzer 2000 (GE Healthcare, Chicago, IL, USA) using a Nikon 20x/0.45 NA Plan Fluor objective, capturing 30 sequential and non-overlapping images of the center of each well. Images were analyzed by Fiji Software and proliferation index determined by counting number of cells with Ki-67^+^ labelling inside the nuclei relative to the total number of nuclei counted.

### 4.11. MSC Differentiation Assay

For MSC characterization, cells were seeded in 6 well-plate at a density of 6000 cells/well and grow until reached 40–60% of confluence. Then, cells were incubated in basal or differentiation MSC media without/with osteogenic (α-MEM 10^−7^ M dexamethasone (Sigma-Aldrich Inc., St Louis, MO, USA), 10^−2^ M β-glycerophosphate and 5 × 10^−5^ M ascorbic acid) and adipogenic (10^−4^ M dexamethasone, 5 × 10^−4^ M IBMX, 10 µg/mL insulin, 10^−4^ M indomethacin) supplements for 21 and 28 days, respectively. Media was changed twice a week and at the end of the osteogenic or adipogenic differentiation, and cells were fixed and stained with Alizarin red or Oil red O, respectively.

For chondrogenic differentiation, a cell pellet of 2 × 10^5^ cells was incubated in 15 mL falcon conical tubes in differentiation media (α-MEM supplemented with 4.5 g/L glucose, 2.5 × 10^-4^ M ascorbic acid, 40 µg/mL l-proline, 100 µg/mL sodium pyruvate, 100 µg/mL ITS, 10^−7^ M dexamethasone, and 10 ng/mL TGF-β3) for 28 days. Then, cell pellet was fixed with paraformaldehyde 4%, processed for histologic analysis, and stained by H&E and Toluidine blue stain. 

For MSC gene expression, cells were cultured in basal conditions for 3 and 7 days, in absence/presence of TNF-α or IL-4 (10 ng/mL and 100 ng/mL). Cells stimulated with osteogenic and chondrogenic media as above were used as positive controls. Cells were collected for RNA isolation and expression of gene markers for osteogenic and chondrogenic differentiation was analyzed.

### 4.12. RNA Isolation and Reverse Transcription-Quantitative Polymerase Chain Reaction (RT-qPCR)

For gene expression analysis, MSC RNA was extracted using TRizol (Invitrogen, Carlsbad, CA, USA) reagent following manufacturer’s instructions, quantified by NanoDrop ND-1000 (ThermoFisher Scientific, Waltham, MA, USA) and integrity assessed by agarose electrophoresis. RNA (900 ng of total RNA) was digested with TURBO DNA-free Kit (Life Technologies, Carlsbad, CA, USA), according to manufacturer’s protocol, and complementary DNA (cDNA) was synthesized with random hexamers (Life Technologies, Carlsbad, CA, USA) and dNTPs using SuperScript III Reverse Transcriptase (Life Technologies, Carlsbad, CA, USA). RT-qPCR was performed in an iQ5 Real-Time PCR Detection System (Bio-Rad Laboratories, Irvine, CA, USA) using cDNA, gene specific primers for osteogenic and chondrogenic differentiation markers (Table 1), and iQ SYBR Green Supermix (Bio-Rad Laboratories, Irvine, CA, USA). Relative gene expression was calculated using the 2^−ΔCT^ method and normalized with the GAPDH reference gene, considering threshold cycles <35.

### 4.13. Statistical Analysis

Data analysis were performed using GraphPad Prism v7.0 software. Normality distribution of data was tested by D’Agostino and Pearson normality test. For weight, paw swelling measurements and arthritis score, 2-way ANOVA followed by Tukey’s multiple comparisons was used. For non-parametric data, when two groups were compared, Mann–Whitney test was performed. To compare multiple unpaired groups, Kruskal–Wallis test was used, followed by uncorrected Dunn’s multiple comparison test. To compare multiple paired groups, Friedman test followed by uncorrected Dunn’s multiple comparisons was performed. Statistical significance was considered whenever * *p* < 0.05; ** *p* < 0.01; *** *p* < 0.001; **** *p* < 0.0001.

## Figures and Tables

**Table 1:** Sequences of primers used for gene expression analysis by RT-qPCR.

Gene	Primer Sequence
ALP	Forward 5′-GACAAGAAGCCCTTCACAGC-3′ Reverse 5′-CTGGGCCTGGTAGTTGTTGT-3′
RUNX2	Forward 5′-CCGATGGGACCGTGGTT-3′ Reverse 5′-CAGCAGAGGCATTTCGTAGCT-3′
SOX9	Forward 5′-CTGAAGGGCTACGACTGGAC-3′ Reverse 5′-TACTGGTCTGCCAGCTTCCT-3′
ACAN	Forward 5′-CTTGGGCAGAAGAAAGATCG-3′ Reverse 5′-GTGCTTGTAGGTGTTGGGGT-3′
GAPDH	Forward 5′-TGCCACTCAGAAGACTGTGG-3′ Reverse 5′-TTCAGCTCTGGGATGACCTT-3′

## Supplementary Materials

Supplementary materials can be found at https://www.mdpi.com/1422-0067/20/21/5436/s1.

## Abbreviations

CIA	Collagen-induced arthritis
MSC	Mesenchymal stem/stromal cells
TNF-α	Tumor necrosis factor-alpha
IL	Interleukin
RA	Rheumatoid Arthritis
FD	Femoral defect
CIA+FD	Collagen-induced arthritis with femoral defect
RT-qPCR	Reverse transcription-quantitative polymerase chain reaction
CII-IFA	Collagen type II-Incomplete Freund’s adjuvant
