# Supplementary material for: The Systemic Immune Response to Collagen-Induced Arthritis and the Impact of Bone Injury in Inflammatory Conditions
Source: Int J Mol Sci. 2019 Oct 31;20(21):5436. doi: 10.3390/ijms20215436 (PMC6862543; doi:10.3390/ijms20215436)
Supplement: Supplementary file 1 [file ijms-20-05436-s001.pdf]

International Journal of
Molecular Sciences

MDPI

Supplementary

The Systemic Immune Response to Collagen-Induced Arthritis and the Impact of Bone Injury in Inflammatory Conditions

José H. Teixeira [1,2], Andreia M. Silva [1,2], Maria Inês Almeida [1], Mafalda Bessa-Gonçalves [1,2], Carla Cunha [1], Mário A. Barbosa [1,2] and Susana G. Santos [1,2,*]

[1] i3S—Instituto de Investigação e Inovação em Saúde and INEB—Instituto Nacional de Engenharia Biomédica, University of Porto, 4200-135 Porto, Portugal; jhteixeira@ineb.up.pt (J.H.T.); andreiamacsilva@gmail.com (A.M.S.); Ines.Almeida@ineb.up.pt (M.I.A.); mafalda.goncalves@i3s.up.pt (M.B.-G.); carla.cunha@ineb.up.pt (C.C.); mbarbosa@i3s.up.pt (M.A.B.)

[2] Department of Molecular Biology, ICBAS—Instituto de Ciências Biomédicas Abel Salazar, University of Porto, 4050-313 Porto, Portugal

Supplementary Material

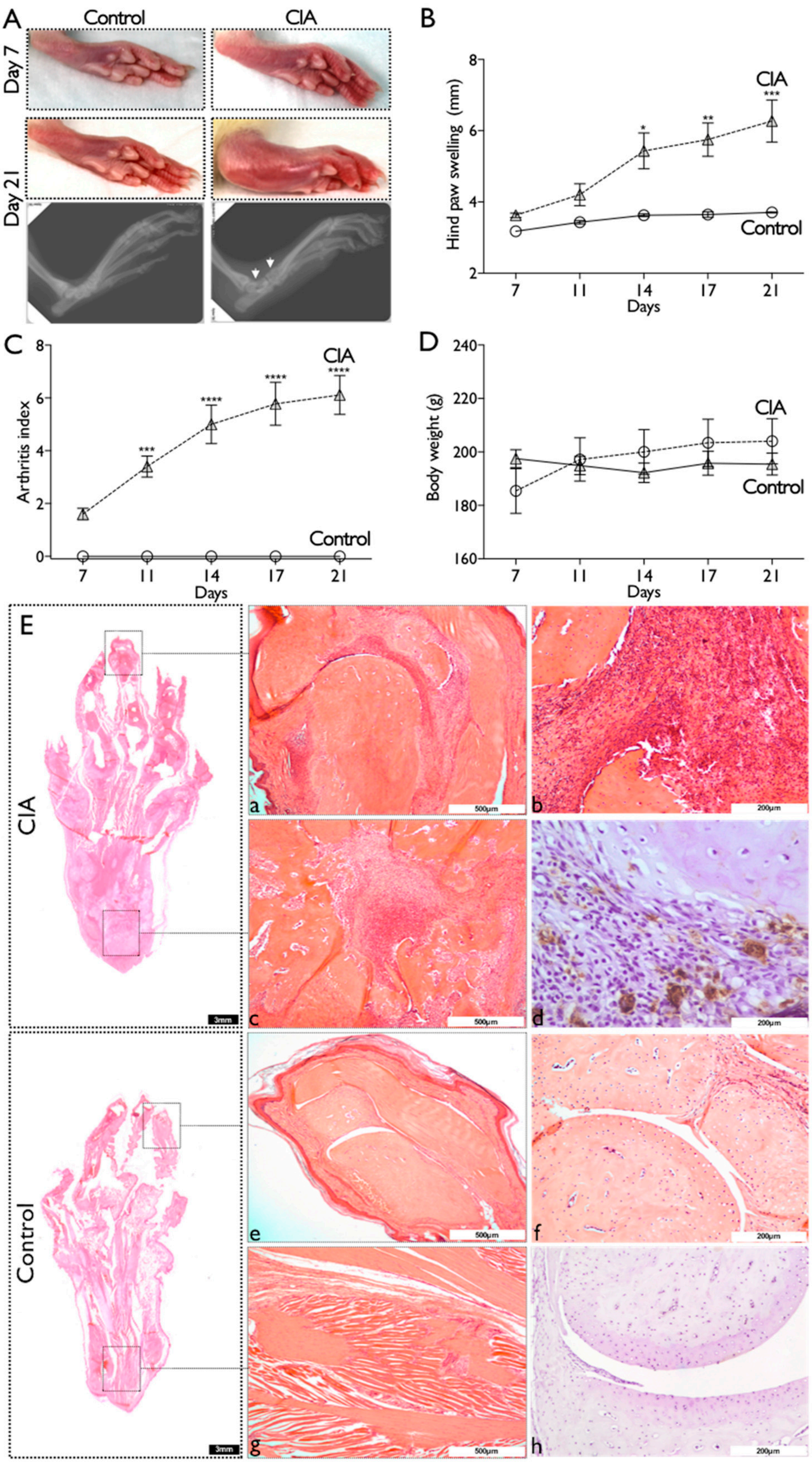

Figure S1. Rat immunization with collagen type II leads to strong hind paw swelling and joint arthritis. A. Macroscopic photographs and X-ray images of animal hind paws at

day 7 and 21 post-immunization. X-ray show density and structural alterations at carpus and radius level (arrows). **B.** Swelling of hind paws was measured along the time after CII-IFA injection and boost. **C.** Representation of clinical arthritis score index based on paw visual inspection during 21 days of animals monitoring. **D.** Weight of CIA and control animals was monitored along the 21 days of animal monitoring. *$p<0.05$, **$p<0.01$, ***$p<0.001$, ****$p<0.0001$ determined by two-way ANOVA test and Turkey's multiple comparisons test. N=4 to 5 animals per group. **E.** Representative hematoxylin and eosin staining of CIA and control animal paw. **E.a.** General morphology of inflamed interphalangeal joint with the synovial space filled by cell infiltrate. **E.b.** Higher magnification showing the extensive infiltration of inflammatory cells and synovial membrane hyperplasia. **E.c.** Adjacent tissue from plantar level showing infiltration of cells, and a generalized paw inflammation. **E.d.** Positive immunostaining for CD68 marker, identifying macrophages in the inflammatory infiltrate. **E.e.** Control rat paws show a normal histologic architecture of joints without cell infiltrates or synovium hyperplasia at (**f**) digital or (**g**) plantar level. **E.h** Immunohistochemistry for macrophage CD68 marker, with no positive cells identified. Scale bar: 3mm (in general morphology of CIA and control paws); 500µm (E.a, E.c, E.e and E.g); 200µm (E.b, E.d, E.f, E.h).

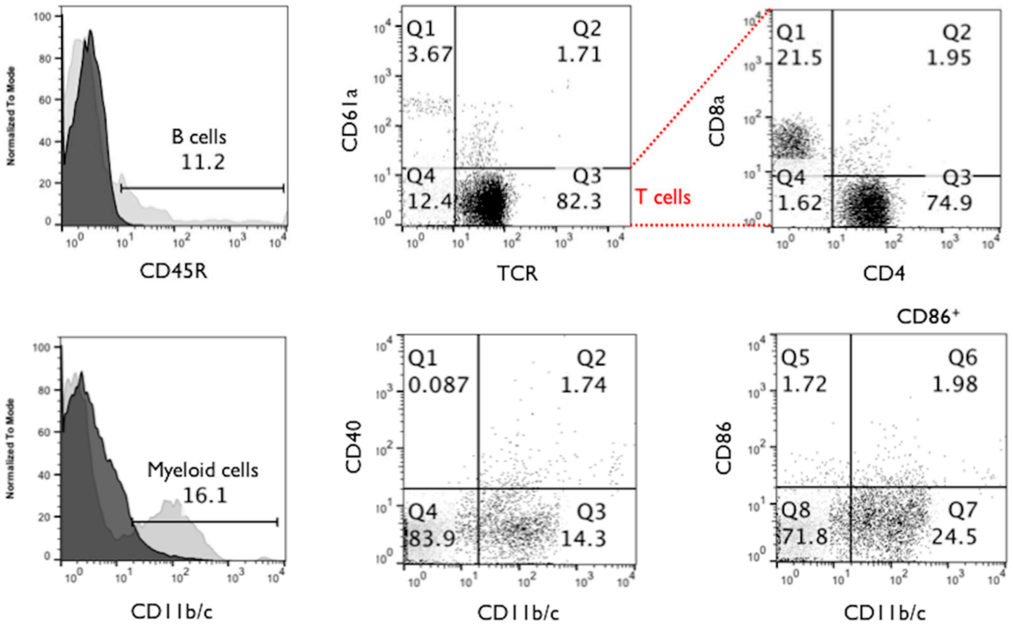

Figure S2. Gating strategy used to evaluate the different immune populations by flow cytometry. Representative plots illustrating the gates and surface markers used to identify: B cells ($CD45R^+/TCR^-$, top right plot); NK ($CD61a^+/TCR^-$) and T cells ($TCR^+/CD61a^-$, top middle plot) and the T cell subsets CD4 ($CD4^+$) and CD8 ($CD8a^+$, top left plot); myeloid cells ($CD11b/c^+$, bottom right plot) and activated myeloid cells ($CD11b/c^+$/ $CD40^+$ and $CD11b/c^+$/ $CD86^+$ bottom middle and left plots). Dark grey histogram represents isotype control. The parent population was defined as cells based on forward and side scatter (FSC/SSC), except for the CD4/CD8 plot, where red lines indicate the quadrant of the parent population.

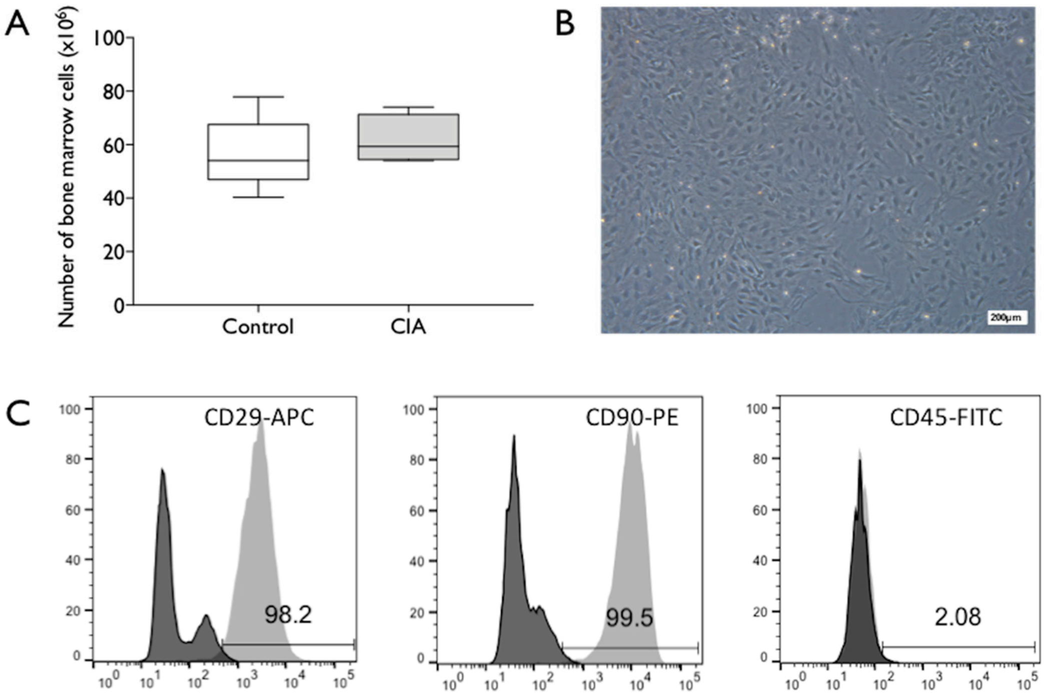

Figure S3. Characterization and immunophenotyping identity of the MSC population isolated from bone marrow.

A. Bone marrow cells were collected by flushing of femurs, counted and cultured. The number of recovered cells is similar between the control and CIA animals. Box plots represent min-to-max distribution of n=4 to 5 animals per group. **B.** Bone marrow-MSC cells were morphologically homogenous with elongated fibroblast-like shape in culture. **C.** Flow cytometry evaluation of cell surface markers CD29, CD90 and CD45 in the isolated MSC populations. The selected population of cells in culture show a high positivity (~98%) for the common MSC markers (CD29 and CD90) and a residual positivity for haematopoietic marker (CD45). Scale bar 200µm.

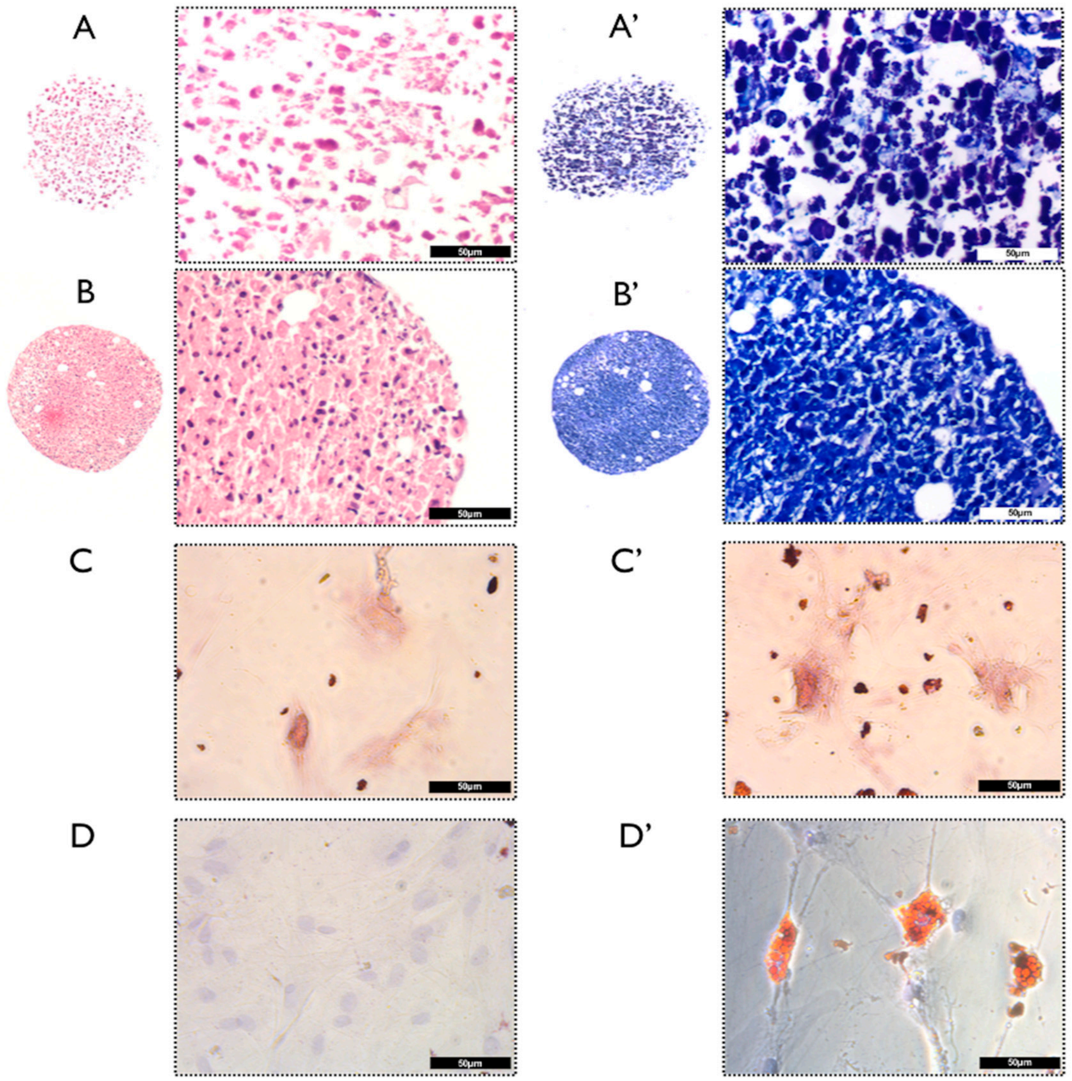

Figure S4. Evaluation of the differentiation capacity of the animals isolated bone marrow-MSC into chondrogenic, osteogenic and adipogenic lineages.

Hematoxylin & eosin staining show a very different pellet size and morphology between the basal (**A**) and chondrogenic stimuli (**A'**) conditions. **B.** In chondrogenic condition the architecture of pellet is more compact with a network between the cells, indicating matrix formation by the toluidine blue staining (**B'**), compared to unstimulated condition (**B**). **C.** The Alizarin red staining showed more mineralized matrix after the osteogenic induction (**C'**), indicating the capacity of BM-MSC to differentiate into osteoblasts. **D.** Oil-red O staining results show red lipid vacuoles after incubation with adipogenesis-stimulating media (**D'**) confirming the capacity of BM-MSC to differentiate into the adipogenic lineage, compared to the basal condition (**D**). Scale bar in all images: 50µm.
